# Machine learning in causal inference for epidemiology

**DOI:** 10.1007/s10654-024-01173-x

**Published:** 2024-11-13

**Authors:** Chiara Moccia, Giovenale Moirano, Maja Popovic, Costanza Pizzi, Piero Fariselli, Lorenzo Richiardi, Claus Thorn Ekstrøm, Milena Maule

**Affiliations:** 1 Cancer Epidemiology Unit, Department of Medical Sciences, University of Turin and CPO Piedmont, Via Santena 7, Turin, 10126 Italy; 2 Department of Medical Sciences, University of Turin, Turin, Italy; 3 Section of Biostatistics, Department of Public Health, University of Copenhagen, Copenhagen, Denmark

**Keywords:** Machine learning, Causal inference, Targeted learning, Doubly-robustness

## Abstract

In causal inference, parametric models are usually employed to address causal questions estimating the effect of interest. However, parametric models rely on the correct model specification assumption that, if not met, leads to biased effect estimates. Correct model specification is challenging, especially in high-dimensional settings. Incorporating Machine Learning (ML) into causal analyses may reduce the bias arising from model misspecification, since ML methods do not require the specification of a functional form of the relationship between variables. However, when ML predictions are directly plugged in a predefined formula of the effect of interest, there is the risk of introducing a “plug-in bias” in the effect measure. To overcome this problem and to achieve useful asymptotic properties, new estimators that combine the predictive potential of ML and the ability of traditional statistical methods to make inference about population parameters have been proposed. For epidemiologists interested in taking advantage of ML for causal inference investigations, we provide an overview of three estimators that represent the current state-of-art, namely Targeted Maximum Likelihood Estimation (TMLE), Augmented Inverse Probability Weighting (AIPW) and Double/Debiased Machine Learning (DML).

**Supplementary Information:**

The online version contains supplementary material available at 10.1007/s10654-024-01173-x.

## Introduction

The advent of advanced technologies and data collection methods has led to an increase in the complexity of modern epidemiological studies, compelling researchers to work with high-dimensional data more frequently. In parallel, the adoption of Machine Learning (ML) techniques has risen, thanks to their ability to learn patterns and relationships from the data, without explicitly programming for every condition.

Until now, ML algorithms in epidemiology have been mostly used to perform prediction tasks, for example in disease diagnosis, patient prognosis, or treatment response [1–3]. ML algorithms excel at learning complex patterns from data, allowing analysts to generate accurate predictions based on the available information. The increasing use of ML in epidemiological research has sparked interest in the context of causal inference, where the goal is to draw causal conclusions on a relationship of interest. In this context, researchers aim to define a causal estimand, representing the quantity they seek to estimate, and then establish the assumptions necessary to express it in terms of observed data through a process known as identification. Thereafter, the focus shifts to estimation and inference tasks. A major risk to causal inference when using observational data is the presence of confounding. Common confounding adjustment techniques include multivariable regression models, propensity score methods, and g-methods [4]. All these approaches typically employ parametric models. However, parametric models rely on correct model specification, which can be particularly challenging in the context of high-dimensional data. For example, in genetic epidemiology, researchers often deal with datasets containing information on thousands of genetic variants, and aim at capturing complex interactions between genetic factors and environmental exposures to understand their combined effect on disease risk. In environmental epidemiology, measuring the joint effects of environmental exposures such as air pollution, water contaminants, and industrial toxins on health outcomes is crucial. Social epidemiology aims at studying determinants of health, often involving a wide range of high-dimensional covariates related to socioeconomic status, such as education, employment, and neighborhood characteristics. In life-course epidemiology, researchers analyse high-dimensional longitudinal data to understand how various exposures and factors affect health outcomes over time throughout life. In these contexts, ML methods that do not require the specification of a functional form of the relationship between variables could unfold their full potential reducing the bias arising from model misspecification.

Over the past decade, a growing research effort has sought to explore how to exploit the excellent predictive performance of ML to address the challenge of establishing causal relationships in epidemiological studies [5–7].

In this article, we aim to give an introduction of estimators that allow the integration of ML in the process of causal effect estimation. We will provide an introduction to ML key concepts, such as supervised learning, hyperparameter tuning, K-fold cross-validation and overfitting. Then, we will give an overview of the statistical model misspecification problem, followed by a description of the methods that allow for the use of ML for the estimation of the Average Treatment Effect (ATE). We will then cover plug-in estimators and emphasise the plug-in bias problem. Finally, we will illustrate three doubly-robust estimators that address plug-in bias, providing an efficient estimate of the ATE.

Since causal inference deals with both observational and randomized control trials, throughout the article “exposure” and “treatment” terms are used interchangeably.

## What is machine learning?

ML techniques have gained increasing popularity in epidemiology thanks to their excellent performance in prediction tasks. These algorithms use data as the “experience” from which to learn and gradually improve their performance, mimicking the human learning task. The distinction between ML and statistical approaches is undefined and the classification of a particular methodology as either “machine” or “statistical” learning often depends on its historical context [8]. The terminology between the two fields differs even if concepts are similar. Bi and colleagues [8] present a useful glossary of ML and statistical/epidemiologic equivalents.

In this article, we will focus on the specific area of ML known as “supervised” learning. Supervised learning works with a dataset where the dependent variable (e.g., presence or absence of a given disease) is observed for each unit/subject, as in standard epidemiological models, and it is named the “label” [8]. Supervised learning automatically and adaptively learns a general rule that maps input (the predictors) to outputs (the label) in the dataset and that can be used to make predictions on new data.

ML model development and evaluation involve three main steps: training, validation, and testing. During the training, various models with different hyperparameter configurations (i.e., parameters whose values control the learning process) are trained on data to learn patterns and relationships between variables. In the validation phase, prediction errors are assessed to select the best-performing model. In the test phase, the generalisation performance of the chosen model is evaluated on unseen data (i.e., the model’s ability to generalise on “out-of-sample” data) [9].

Typically, K-fold cross-validation is used as a procedure of data partitioning that repeats the training and validation phases on the same data. The procedure randomly divides the observations into K groups, named folds. K-1 groups are used to fit the model that is subsequently validated on the previously excluded fold. The procedure is repeated K times, each time excluding one of the different folds. The K final estimates of the metric used for evaluation, e.g. the mean squared error (MSE), are then averaged, to produce a single, robust, measure of model performance on training data.

To enable the model to “learn” and refine its parameters, many ML algorithms perform an iterative optimization process to minimise or maximise an objective function that captures the overall learning task. During the training phase, a function (for example, a metric like the above-mentioned MSE) quantifies how far the predicted value is from its observed value, guiding the optimisation process.

A central problem of many data analyses is finding the right balance between model flexibility and simplicity. This is crucial for achieving an optimal trade-off between bias and variance. The bias is the difference between the mean value of the model-predicted parameter and its true value. The variance reflects the model sensitivity to small fluctuations in the training set. Large bias results in an underfitted model (a model that is too simple and fails to capture the underlying patterns), whilst large variance results in an overfitted model (a model that fits the training data closely, also memorising random fluctuations in the training set).

Different methods employ different strategies to reach the optimal bias-variance trade-off. For example, parametric models achieve the balance by assuming specific data distributions and limiting the number of parameters (usually substantially smaller than the number of parameters in ML modelling). However, their assumptions may limit the ability to capture complex relationships in the data. Conversely, increasing the number of parameters relaxes these constraints, affording more flexibility and guarding against bias from model misspecification. However, this flexibility can lead to wider confidence intervals, reflecting increased variance [10]. Regularisation methods such as lasso, ridge, and elastic net constrain the model flexibility using a penalization factor. By penalizing coefficients in the model, these techniques reduce the risk of capturing noise in the training data to achieve more accurate predictions while promoting generalisation to new data. Lasso penalizes the absolute values of the coefficients, often shrinking some coefficients to zero, thus performing feature selection. Ridge penalizes the squared values of the coefficients, which tends to shrink the coefficients uniformly and is particularly effective when dealing with multicollinearity. Elastic net combines the penalties of both lasso and ridge, balancing between feature selection and coefficient shrinkage. Furthermore, ML models use appropriate validation and tuning stages to reach the best bias-variance trade-off and to avoid overfitting.

### SuperLearner

The SuperLearner is a generalisation of stacking methods [11], a technique in which many models are used and weighted to produce, as output, a new model. It uses cross-validation to estimate the performance of multiple supervised learning models. The collection of ML and parametric models considered by SuperLearner can be large, and the models may differ in terms of how they work (mathematical functions used to make predictions), how they measure the model ability in predicting the expected outcome (loss function), how they explore the solution space (searching algorithm) [12]. SuperLearner can include more structured methods, like parametric models or lasso, and less structured ones, like random forest, support vector machine, and neural network (see [8] for an introduction to these algorithms). The weighted average used in the SuperLearner solves the critical challenge of the a priori selection of a single algorithm [12]. As a consequence, the SuperLearner performs asymptotically at least as well as the best choice among all possible weighted combinations (finite sample oracle inequality theorem [12]), and can capture a wider range of data patterns, making more reliable predictions in different contexts [12].

## Causal research

Causal research can be generally divided into two approaches: confirmatory and exploratory [13]. The main goal of the confirmatory approach is the evaluation of the evidence, relying on a-priori knowledge, and assuming, as a starting hypothesis, a causal structure describing the relationships between the variables involved (e.g., using directed acyclic graphs (DAGs)). Data analysis is then performed to confirm or not the starting hypothesis. The exploratory approach, on the other hand, does not start with a priori hypotheses. Instead of specifying a model prior to data analysis, it aims at stimulating the exploration of alternative hypotheses and infers the causal model directly from the data. A branch of causal methods named causal discovery has been developed to be used for this purpose, exploiting the power of ML [14]. In this article, we will focus on methods that integrate ML for causal effect estimation in the confirmatory approach.

### The problem of model misspecification

The use of parametric models is very popular thanks to their simplicity and useful asymptotic properties that allow the construction of confidence intervals and hypothesis testing [7]. As the sample size increases, the central limit theorem and the law of large numbers may be used to reach desirable properties: efficiency^1^, consistency^2^ and asymptotic normality^3^ [7]. However, for the estimator to converge in probability to the true parameter value (i.e. to be consistent), and to gain other desirable asymptotic properties, it is assumed that the underlying model is correctly specified. In practice, however, parametric models are often misspecified and, consequently, they cannot optimally capture the true data-generating process. One of the strong and often unverifiable assumptions parametric models rely on is the correct model specification of the exposure-outcome relationship. If this assumption is unmet, the estimate can suffer from “estimation bias”^4^ [15]. To specify a parametric model correctly, it is necessary (i) to assume that the true data-generating process belongs to a specific parametric family (in this way, specifying correctly the link function, a possibly nonlinear relationship can be mapped into a linear one), (ii) to include a correct set of exposure-covariates and/or covariate-covariate interactions, if any, and (iii) to model potential nonlinearities appropriately [16]. An example is the use of a logistic regression to estimate the propensity score: it restricts the type of relationship between exposure and confounders, assuming that the log-odds of exposure are appropriately described by a linear combination of the covariates [16].

Classical statistical theory often ensures that the estimator, obtained with the maximum likelihood estimation, is asymptotically efficient, i.e. that it achieves the lowest possible variance among all consistent estimators in large samples, under certain regularity conditions (smoothness). This optimality holds when the assumed parametric model is correct and the sample size is large. As a result, while parametric approaches offer simplicity and computational efficiency, they may not adequately capture the complexity of real-world data, because the assumption on the underlying distribution is often too restrictive.

Nonparametric or semiparametric methods do not rely on assuming that the data follow a specific parametric distribution indexed by finite-dimensional parameters. Nonparametric models are particularly useful when there is limited knowledge or assumptions about the underlying exposure mechanism, outcome mechanism, or both. Despite the absence of parametric assumptions, nonparametric models can achieve convergence rates, and valid confidence intervals (CIs) can be constructed even when ML techniques are used to handle high-dimensional data and capture complex relationships between variables.

In recent years, estimators for causal effects that exploit ML efficiency have been developed [17]. These methods join forces of the two, apparently distinct, perspectives of causal inference and ML, so that each one can take advantage of the other. The integration of ML methods in estimators for a causal effect can mitigate the assumption of correct model specification thanks to their flexibility and capability to approximate complex functions, to handle interactions and nonlinearities, and avoiding functional-form restrictions [7, 8].

### Definition of the causal framework

According to the counterfactual theory of causation [18], questions about the causal effect of an exposure A on an outcome Y in a particular population can be expressed in terms of counterfactual contrasts. A counterfactual is a ‘what-if’ statement that describes what would have happened in the target population under different exposure levels than those actually observed. A key causal estimand is the average treatment effect (ATE) that, for a binary exposure, represents the difference between the expected value of the outcome that would have occurred under exposure A = 1 (exposed) and the outcome that would have occurred under exposure A = 0 (unexposed) (the so-called potential outcomes). Mathematically, it is defined as:

ATE = E[Y(1) − Y(0)]

where E denotes the expectation, and Y(1) and Y(0) are the potential outcomes under A = 1 and A = 0, respectively.

**Fig. 1:**
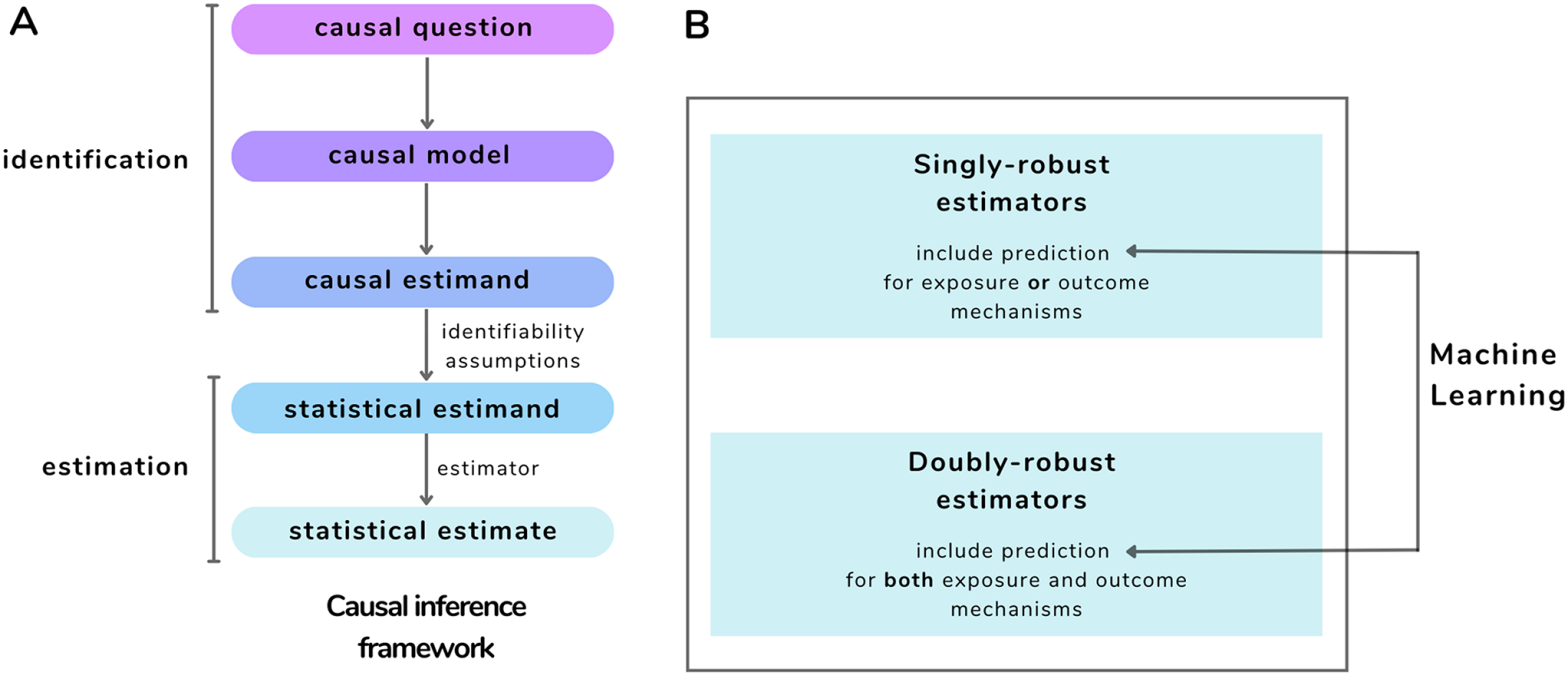
Visual synthesis of the article. In A, the different steps of a causal inference framework. In B, estimators for causal effect that integrate Machine Learning methods, bridging the gap between statistical inference and Machine Learning

To estimate the ATE from observed data, several critical steps and assumptions must be considered within a formal causal framework, such as for example the Causal Roadmap [19], Fig. 1A: (i) after the identification of the research question and (ii) the specification of the causal model (e.g., through a DAG) representing the assumed relationships between variables, (iii) the research question is translated into the causal estimand of interest (e.g. the ATE). To make the causal estimand quantifiable from the observed data, (iv) it is translated into a statistical estimand. However, to establish a causal interpretation of the statistical estimand, it is essential to ensure that the following identifiability assumptions are met [15, 19]:

**Counterfactual consistency**: The observed outcome is consistent with the potential outcomes under the observed exposure level.

**No interference**: The potential outcomes for an individual are not affected by the exposure status of other individuals.

**Exchangeability**: The distribution of potential outcomes is the same across exposed and unexposed, given the covariates.

**Positivity**: There is a non-zero probability of receiving each level of the exposure for all levels of covariates.

After evaluating the assumptions encoded in the causal model and ensuring adequate data support, v) the statistical parameter can be estimated.

### Statistical estimators of the ATE

In this article, we focus on the estimation of the ATE. The Risk Difference (RD) is a straightforward measure of the ATE (for continuous or binary outcome). However, packages implementing the methods illustrated here are versatile and capable of providing estimates of the treatment effects also on the risk ratio and the odds ratio scales (for binary outcomes). Furthermore, they are able of accommodating other causal estimands beyond the ATE, such as the Average Treatment effect among the Treated (ATT) and among the controls (ATC) [20–22] and a variety of structural models as detailed in Table 1 [21, 22].

To estimate the ATE, causal inference approaches typically involve fitting “nuisance models” [7] to the data before the final parameter estimation step. These nuisance models aim to estimate the conditional expectation of the outcome given exposure and confounders (outcome mechanism) and/or the conditional probability of exposure given the confounders, namely the propensity score (exposure mechanism).

Traditionally, “nuisance models” are fitted using parametric models. When the number of confounders is high-dimensional and exceeds the sample size, traditional parametric models have a high probability to be misspecified [10]. Since nuisance models are purely predictive problems that do not involve causal interpretation [23], they can benefit from the use of methods with high predictive ability and particularly suited to work with high-dimensional data, such as ML. Supervised ML techniques, like decision trees, random forests, support vector machines, neural networks, and ensembles like the SuperLearner are particularly suited for the purpose [19, 24]- [26].

### Plug-in estimators of the ATE

The predictions obtained from the nuisance models with a single ML method, or with the SuperLearner, can be integrated into the estimator for the ATE (Fig. 1B). An example are plug-in estimators, statistical estimators where estimates of specific quantities, such as parameters or functions, are plugged into a predefined formula to compute the estimate of interest. Two examples are Inverse Probability Weighting (IPW) and g-computation: they involve plugging in estimated quantities (propensity score (PS) in the case of IPW, and potential outcomes in the case of g-computation) into a specific formula to estimate the ATE. They are “singly robust” estimators because they rely on the correct specification of one nuisance model, either the one representing the exposure mechanism (e.g., for the IPW), or the one representing the outcome mechanism (e.g., for the g-computation) [24].

The PS, the nuisance model for the exposure mechanism in IPW, aims at reducing the information from all confounders in one parameter, the “propensity” to be exposed to the exposure of interest, allowing for an optimal balance of observed covariates between exposed and unexposed. The PS can then be used to control for confounding in different ways. For example, it can be integrated in the IPW estimator for the causal parameter of interest: each observation is weighted with the inverse of the probability, conditional on all confounders, that an individual received the exposure that they actually received. The probability is 1/PS for exposed and 1/(1-PS) for unexposed individuals. The weights serve to create pseudo-populations where the exposure status no longer depends on the confounders.

G-computation, on the other side, is an example of an estimator that requires a nuisance model for the outcome mechanism. In this case, the potential outcomes, treated as a missing data problem, are predicted from the model for the outcome. Potential outcomes are then plugged in the g-computation estimator to obtain an estimate for the ATE.

ML techniques can replace the use of parametric models for the computation of the PS and the potential outcomes, improving the quality of the prediction.

However, the potential advantages of using ML to estimate the nuisance models in plug-in estimators come at a price and involve challenges associated with increased complexity, overfitting, sample size requirement and, especially, the risk of (plug-in) bias. The reason is that ML methods are solving an optimization problem for the prediction of the nuisance models, but the bias-variance trade-off they reach may be suboptimal for the task of interest, i.e., obtaining an unbiased estimate of the ATE [12]. Additionally, when integrating nonparametric models, plug-in estimators typically exhibit bias larger than $\:\frac{1}{\sqrt{n}}$, where *n* is the sample size^5^, and experience slower convergence rates compared to parametric methods. This phenomenon is known as the curse of dimensionality, and it implies that exponentially larger sample sizes are required to obtain parameter estimates that are as close as possible to the true parameter values [24, 27]- [29].

### Doubly-robust estimators

In response to the limitations of plug-in estimators, doubly-robust estimators [24] have been proposed. They achieve useful asymptotic properties, including the construction of valid confidence intervals also when nuisance models are estimated using ML [19, 30].

They are called doubly-robust because they provide two opportunities to obtain an unbiased estimator of the ATE. Similarly to singly-robust estimators, they also require predictive steps before the effect estimation but, in this case, two separate nuisance models, one for the exposure and one for the outcome mechanism, are obtained (Fig. 1B). After the prediction of propensity and outcome models, the two nuisance models are combined for the estimation of the target causal effect. Such an estimator will be consistent if either the propensity or the outcome model is specified correctly, but not necessarily both [30]. However, the asymptotic efficiency and the ability to perform standard parametric-rate inference (e.g. with rates of convergence typically associated with parametric models) on the target parameter can be achieved only if both nuisance models are specified correctly [30].

The advantage of the use of ML in conjunction with doubly-robust estimators is the ability of doubly-robust estimators to achieve small bias more readily than singly-robust, owing to the mathematical properties of their estimation error. Specifically, the bias is less than $\:\frac{1}{\sqrt{n}}$, where *n* is the sample size, if the errors in both the nuisance models are substantially smaller than $\:\frac{1}{\sqrt[4]{n}}$, condition that ML estimators can satisfy under smoothness and sparsity assumptions [10].

It is important to be cautious, as it has been shown that doubly-robust estimators are generally less efficient than those obtained with correctly specified parametric models based on maximum likelihood estimation [17]. Moreover, if both nuisance models are misspecified, the resulting estimate may exhibit larger bias than the one obtained with a single, misspecified maximum likelihood model [24]. However, while parametric models may converge faster and require smaller sample sizes to achieve a certain level of efficiency, they may not necessarily exhibit higher accuracy compared to ML models, which typically offer greater flexibility and may capture more complex relationships within the data.

To ensure statistical validity of confidence intervals, doubly robust ML estimators require sample splitting and cross-fitting. Sample splitting involves dividing the study population into estimation and training samples. The training sample is used for training ML algorithms to estimate nuisance models, while the estimation sample is employed for estimating the ATE. This yields a doubly-robust estimate of the ATE, derived from a random half of the study population. However, the resulting confidence intervals tend to be wider than those obtained using the entire sample due to halving the sample size. To mitigate this issue and regain some of efficiency, cross-fitting involves repeating the estimation procedure multiple times using different subsets of the data for training and estimation. Averaging the estimates obtained from these different subsets reduces variability in the estimates, yielding more precise estimates of the treatment effect.

In the next three sections, we will discuss the three most commonly used doubly-robust estimators: Augmented Inverse Probability Weighting (AIPW), Double/Debiased Machine Learning (DML) and Targeted Maximum Likelihood Estimation (TMLE). We will explore their conceptual details, principles, advantages and examples of applications. In Table 1, for each explored method, relevant theoretical articles, tutorials, worked examples, reviews and software are listed.

#### Augmented inverse probability weighting and double/debiased machine learning

AIPW, first proposed by Robins and colleagues [32] and further developed by Scharfstein and colleagues [33], is a doubly-robust estimator based on the estimating equation methodology [12]. As in IPW, the basic idea is to use weights to adjust for differences in the distribution of confounders between exposed and unexposed. To obtain the AIPW estimator, the IPW estimator is augmented by a term that involves the outcome regression. The augmentation term is the weighted average of the two potential outcomes [34] and serves: (1) to increase the efficiency, resulting in a smaller variance than that of the IPW estimator [35], and (2) to provide the estimator with the double-robustness property [36].

If the PS is well specified, then the AIPW estimator simplifies to the IPW estimator. Conversely, if the PS is misspecified, the AIPW estimator reduces to the outcome model [36].

AIPW, derived from the semiparametric efficiency theory, maintains the double robustness property even when combined with ML techniques [28].

AIPW serves as the foundation for the broader Double/Debiased Machine Learning (DML) framework. In its full-sample implementation, AIPW uses data from all individuals to estimate both the PS and the outcome model, along with the final ATE estimate. However, this full-sample approach carries the risk of introducing correlation between the nuisance models and the final ATE estimate, potentially impacting performance in unpredictable ways [10].

To address this limitation, the DML framework [28], proposed in 2018 by Chernozhukov and colleagues, builds upon AIPW by incorporating sample splitting and cross-fitting techniques. Splitting the sample into two parts, one to estimate the nuisance parameters and the other to compute the final ATE estimate, reduces the risk of bias of the full-sample estimator. Moreover, sample splitting helps to mitigate overfitting bias, allowing for the use of various ML methods such as lasso, random forests, and neural networks, depending on the data characteristics and problem at hand. In DML, ML methods are used to predict, separately, the outcome Y and the exposure A from the covariates. The predictions are then combined by regressing the residuals of Y on the residuals of A, guided by an estimating equation^6^ that ensures double robustness [22, 28, 37], overcoming the problems of plug-in estimators. DML is particularly suited to settings with a large number of covariates [28]. The authors of DML provide guidance on selecting the appropriate ML methods [28] based on the specific characteristics of the data and the problem at hand.

#### Targeted maximum likelihood estimation

TMLE is a doubly-robust, maximum-likelihood–based estimation method, developed by van der Laan and Rubin [31]. In addition to the initial estimation of the outcome and exposure models, TMLE involves a “targeting” step to get the best estimate of our target parameter of interest (e.g., ATE) [38].

To give some insights into how the method works, an example is provided illustrating the technical steps involved in using TMLE to estimate the ATE of a binary exposure A on an outcome Y, adjusted for baseline confounders W:

##### Prediction of the outcome model

In the first stage, the conditional expectation of the outcome given exposure and covariates $\:Q=E\left(Y\right|A,W)$ is modelled and used to predict every individual’s outcome. Such a model can be fitted using the SuperLearner. We can then obtain an estimate of ATE based on g-computation. However, this estimate is singly-robust (thus, susceptible to bias): it is based on a correct estimate of $\:Q$ rather than the estimate of the ATE.

##### Prediction of the propensity score

To overcome this problem, information on the exposure mechanism is used. The PS, $\:P(A=1|W)$, is estimated, for example, using the SuperLearner.

##### The clever covariate and estimation of the fluctuation parameter ε

The PS is used to create a variable, named clever covariate, defined as $\:\frac{1}{P(A=1|W)}$ for the exposed individuals and $\:\frac{-1}{P(A=0|W)}$ for the unexposed individuals. The clever covariate is crucial in updating the initial outcome estimates using information on the exposure and to optimise the bias-variance trade-off for the target parameter (e.g., the ATE) rather than for $\:Q.$ A predefined regression model is used to update the initial outcome estimates: the observed outcome $\:Y$ is regressed on the clever covariate as the only predictor, with the initial obtained outcome prediction *Q*, as a fixed intercept. The regression coefficient ε that will be estimated, based on maximum likelihood estimation, is called the fluctuation parameter. By solving an estimating equation (which sets the efficient influence function equal to zero (see Supplementary Material)) [11], the clever covariate ensures that the estimator becomes approximately unbiased and gains useful asymptotic properties [12].

##### Updating of the outcome model

The fluctuation parameter is then used to update the initial estimate of $\:Q$, yielding the two final potential outcomes. The ATE is then computed as the average difference between the two updated potential outcomes across individuals.

The literature on TMLE is expanding [38], and this technique is becoming the most widely used doubly-robust approach [50–52]. A recent systematic review examined the increasing adoption of TMLE in public health and epidemiological studies, on a wide range of research questions and outcomes [38]. The diverse applications of TMLE highlight the variety of complex causal effect estimation problems where this method can show its potential, such as multiple time point interventions, longitudinal data, post-intervention effect modifiers, dependence of the exposure assignment between units or censoring, causally connected units, hierarchical data structures, randomisation at the cluster level, large electronic health record data, and meta-analyses [38].

Practical guidelines and tutorials have been published on the implementation of TMLE to model the effects of a binary exposure [11, 37] and sequential interventions with time-varying confounders [38]. These resources offer valuable insights into applying TMLE methodology in various research settings.

#### Comparison between AIPW and TMLE

Since TMLE and AIPW are based on the efficient influence function (see Supplementary Material), both are mathematically efficient and exhibit similar asymptotic properties. However, while both estimators perform well in large sample settings, they behave differently in finite sample settings with AIPW estimates subject to larger variability than TMLE estimates [30]. An important difference between TMLE and AIPW is that they are both estimating-equation-based estimators, but the former is also a loss-based estimator that makes use of the maximum likelihood estimation. Estimating-equation-based methodology aims at providing estimators with minimal asymptotic variance, without imposing constraints to ensure that the estimated values are realistic and feasible within the context of the observed data [12]. AIPW has the same weaknesses of IPW when it comes to the positivity assumption and unstable weights. Under dual misspecification and near-positivity violations, it has been shown that AIPW performs worse than TMLE, and it is unstable when values of the PS are close to zero [12]. On the other hand, AIPW can be relatively easier to implement, as it does not involve the iterative updating of models, and might require fewer computational resources compared to TMLE.

**Table 1:** List of relevant theoretical articles, tutorials, worked examples, reviews and software for AIPW, DML and TMLE

	Relevant articles	Tutorials	Worked examples	Reviews	Software
AIPW	Robins et al. 1994 [32]	Kurz 2022 [36], Smith et al. 2022 [39]	Papini et al. 2022 [40], Tseng et al. 2023 [41]	-	https://cran.r-project.org/web/packages/AIPW/index.html designed specifically to estimate the ATE of a binary exposure
DML	Chernozhukov et al. 2018 [25]	Bach et al. 2024 [22]	Gon et al. 2022 [42], Shinkawa et al. 2022 [43]	-	https://cran.r-project.org/web/packages/DoubleML/index.html designed to estimate ATE, ATT, ATC, treatment effect heterogeneity, a variety of structural models (Partially Linear Regression (PLR), Partially Linear Instrumental Variables (PLIV)) Instrumental Variable Models (IVM), Interactive Models (IRM), and Instrumental Interactive Models (IIVM))
TMLE	Van der Laan & Rose 2011 [12], Van der Laan & Rose 2018 [44]	Luque-Fernandez et al. 2018 [45], Smith et al. 2022 39], Schuler & Rose 2017 [11]	Pang et al. 2016 [46], Kreif et al. 2017 [47], Veit et al. 2020 [48], Izano et al. 2019[49], Chavda et al. 2022 [50], Kang et al. 2021 [51], Lim et al. 2019 [52], Luque-Fernandez et al. 2018 [53]	Smith et al. 2023 [38]	https://cran.r-project.org/web/packages/tmle/index.html designed to estimate ATE, ATT, ATC, marginal structural model for a binary point treatment effect and effect stratified by a binary mediating variable

## Applications in epidemiological studies

The predictive power of ML can be exploited in the prediction steps of causal effects estimators. In this paper, we have illustrated three currently available doubly-robust estimators that integrate ML in the estimation process. In particular, doubly-robust estimators that exploit ML are especially promising for causal questions as they help relaxing the model misspecification problem, still providing efficient and unbiased estimates of the target parameter. Doubly-robust methods that include SuperLearner in the estimation process were applied in various epidemiological domains, serving distinct purposes, such as risk factors identification, treatment effect estimation, evaluation of effectiveness of intervention, heterogeneous treatment effect, research on social determinants of health. TMLE, in particular, has been applied in non-communicable disease epidemiology, behavioural epidemiology, pharmaco-epidemiology, biomarker epidemiology, environmental epidemiology and occupational epidemiology.

Applying doubly-robust methods alongside traditional techniques is beneficial since each method is based on distinct assumptions. By employing a variety of approaches, researchers can gain deeper insights into the robustness of their findings and assess the validity of underlying assumptions. This enhances the credibility and reliability of research findings.

Luque-Fernandez and colleagues [45] conducted a motivating example aiming to demonstrate with a simulation study the advantage of double‐robustness. They estimated the 1-year mortality risk difference and odds ratio of death for cancer patients treated with monotherapy (radiotherapy only) versus dual therapy (radiotherapy and chemotherapy). They compared the performance of different estimation methods, including naïve regression, AIPW, and three variations of TMLE. In TMLE-1 authors used logistic regressions to model the exposure and the outcome mechanism, in TMLE-2 they used SuperLearner with the default library and in TMLE-3 the SuperLearner with user‐supplied library. To simulate real-world scenarios, researchers intentionally introduced mild misspecifications in the treatment and outcome models, such as omitting interactions between age and comorbidities in logistic regression models. Additionally, they ensured that the data generation process often resulted in near-practical positivity violations, where certain subgroups rarely or never received treatment.

Their findings showed that TMLE methods, especially TMLE-2 and TMLE-3 involving Super-Learner libraries, performed better than naïve and AIPW approaches when treatment and outcome models were misspecified. The true ATE was 19.3% and the marginal odds ratio (MOR) of monotherapy versus dual therapy was 2.5. The naïve approach overestimated the MOR by 24%, whereas the AIPW and TMLE-1 overestimated it by 20%, likely because of model misspecification. TMLE‐3, which used a more diverse SL library, reduced the bias for the MOR to 12%. Regarding the simulation results for the risk differences, the AIPW estimator overestimated the ATE by 7%, whereas TMLE‐1 overestimated it by just 3%. TMLE‐2 and TMLE‐3 reduced the bias for the ATE to 0%.

Moreover, they demonstrated the double-robustness property of TMLE by running a second set of simulations with correctly specified propensity scores and with the outcome model incorrectly specified. The true ATE was 22.4% and the MOR of monotherapy versus dual therapy was 2.6. The naïve approach overestimated the MOR by 11%, whereas the AIPW, TMLE-1 and TMLE‐2 overestimated it by 7%. TMLE‐3, which used a more diverse SL library, reduced the bias for the MOR to 4%. Regarding the simulation results for the risk differences, the AIPW and the TMLE‐1 estimator overestimated the ATE by 1%, while TMLE‐2 and TMLE‐3 reduced the bias for the ATE to 0%. Another example of a real-data application in which the use of doubly-robust methods yields results that diverge from those obtained through standard analytical approaches is the study by Schnitzer and colleagues [54], aimed at estimating the differences in the marginal expected number of gastrointestinal infections under different durations of breastfeeding. They employed various estimation methods to address baseline and time-dependent confounding, including G-computation, TMLE with parametric modelling, TMLE with Super Learner, and a stabilized IPW estimator. Results from the different methods revealed a consistent trend: longer durations of breastfeeding were associated with reduced infection rates. However, the magnitude of this effect varied across methods. For instance, in the comparison between breastfeeding durations of 3–6 months versus 1–2 months, the estimates ranged from − 0.021 (− 0.042, 0.000) for IPW to -0.039 (− 0.062, − 0.016) for TMLE with Super Learner. Similarly, in the comparison between breastfeeding durations of 9 + months versus 3–6 months, the estimates varied from − 0.013 (− 0.020, − 0.005) for IPW to -0.024 (− 0.038, − 0.010) for TMLE with SuperLearner.

However, there are also examples in the literature in which different methods did not lead to remarkably different results. In the cohort study by Ehrlich and colleagues [55], researchers used doubly-robust methods, in particular TMLE, to investigate the causal relationship between exercise during the first trimester of pregnancy and infant size at birth. The study, conducted among 2,286 women receiving care at Kaiser Permanente Northern California, estimated the differences in the risk of delivering infants who were small or large for gestational age (SGA or LGA, respectively) based on exercise habits during pregnancy. Inferences from TMLE were compared with those from the IPW estimator. Results were similar using IPW and TMLE. Exercise at the cohort-specific 75th percentile was associated with an increased risk of SGA births. There was a slight difference in the TMLE and IPW estimates for performing any amount of vigorous intensity exercise versus none, particularly among underweight and normal-weight women. For these, IPW results indicated a risk difference for delivering SGA neonates of 0.0418 (− 0.0113, 0.0949), while the TMLE estimate was lower: 0.0294 (− 0.0107, 0.0695).

In another study by Kreif and colleagues [47], longitudinal TMLE, IPW and g-computation were compared to evaluate the impact of nutritional interventions on clinical outcomes among critically ill children in a United Kingdom study. The likelihood of a child being discharged alive from the pediatric intensive care unit (PICU) by a specific day was measured, considering a spectrum of static and dynamic feeding protocols. Statistical methods produce similar results. For example, the probability of discharge by the end of day 5 for the “feed from day 3” regime was estimated to be 0.54 (95% CI: 0.47, 0.60) using IPW, 0.59 using g-computation and 0.53 (95% CI: 0.48, 0.59) using TMLE.

While some studies may show no differences in results across different estimation methods, the possibility of estimation bias remains unpredictable. This uncertainty highlights the importance of employing doubly-robust methods to ensure more reliable estimates of causal effects across various study contexts. In particular, when dealing with high-dimensional data, doubly-robust methods offer a powerful framework. They provide a double protection against model misspecification and ensure asymptotic efficiency, making them especially suitable for complex datasets where traditional methods might struggle.

Papadopoulou and colleagues [56] used TMLE to investigate the role of diet as a source of exposure to environmental contaminants in blood and urinary samples in mother-child pairs from six European birth cohorts. Results indicate that higher fish consumption, both in mothers and children, is associated with elevated levels of certain contaminants such as PCBs, PFAS, mercury, and arsenic. Conversely, organic food consumption during childhood is linked to lower levels of pesticide metabolites. This study paves the way to the exposome [57, 58], a paradigm in which information about a multiplicity of exposures over a lifetime is considered together, including external environmental exposures (e.g. air pollution, noise, climate), internal exposures (e.g., blood concentration of chemical products), high-throughput omics layers (e.g., genomics, proteomics), and high-resolution measurements of physical status (e.g., smart devices, watches) [59]. While studies employing TMLE to investigate the full exposome are currently limited, there is potential for this methodology to contribute valuable insights when exploring relationships using a high-dimensional set of environmental exposures.

Another high-dimensional setting where doubly-robust methods with ML have been applied is molecular epidemiology, in the attempt to solve various research questions: the search for sets of candidate biomarkers for a given outcome, to rank the contributions of candidate biomarkers, to measure variable importance, and to reduce the dimensionality with gene expression data [60]. In this context, TMLE-VIM, an extension of TMLE for dimension reduction based on the variable importance measurement, has been proposed. This approach not only takes advantage of the prediction power of ML algorithms, but also accounts for the correlation structures among variables.

## Conclusion

In summary, the implementation of causal estimands using doubly robust ML estimators offers significant advantages for epidemiological research. These estimators are resilient to model misspecification, flexible in handling high-dimensional data, and efficient in providing precise estimates. Additionally, they can accommodate a variety of causal estimands.

## Electronic supplementary material

Below is the link to the electronic supplementary material.

Supplementary Material 1

## Footnotes

1 Efficiency ensures that the estimator is the one with the lowest possible variance (e.g., achieving the Cramér-Rao lower bound).

2 Consistency guarantees that the estimator converges in probability to the true parameter.

3 Asymptotic normality causes the estimator to converge to a normal distribution as the sample size becomes infinitely large.

4 It is important to distinguish between identification bias and estimation bias. The estimation bias is the difference between the estimate obtained from data and the causal estimand. It relates to issues in estimating the causal effect from data, which may arise due to various statistical challenges. It can be solved with better estimation methods, and it is what we will address in this article. Identification bias is the difference between the causal estimand and the causal effect that we aim to measure. It can only be addressed with a better causal model and it is unrelated to the statistical methods used in the analyses (e.g., identification bias pertains to the adequacy of the causal model in representing the true causal relationships) [7]. Throughout the article, with the term “bias” we will refer to the estimation bias and with the concept of “model misspecification problem” to its statistical misspecification.

5 In statistical theory, 1/√n often serves as a benchmark for the expected magnitude of the standard error of an estimator, where *n* is the sample size. This threshold represents the standard deviation of the estimator, indicating the typical variability of the estimator around the true parameter value.

6 Neyman orthogonal moment function.
