# Supplementary material for: Machine learning in causal inference for epidemiology
Source: Eur J Epidemiol. 2024 Nov 13;39(10):1097–108. doi: 10.1007/s10654-024-01173-x (PMC11599438; doi:10.1007/s10654-024-01173-x)
Supplement: Supplementary file 1 — Supplementary Material 1 [file 10654_2024_1173_MOESM1_ESM.docx]

**Supplementary Material**

**Efficient non parametric parameter estimation**

Some estimators reach their target parameter more accurately than others. To assess the characteristics of an estimator, the rate of convergence is used as a measure of how quickly the estimator approaches the true parameter as the sample size increases. Faster rates of convergence imply that the estimator provides more precise estimates with smaller sample sizes. This leads to narrower confidence intervals, which means more accurate inferences about the population parameter using smaller amounts of data. In contrast, slower rates of convergence require larger sample sizes to achieve the same level of precision[1].

The lower bounds of estimation error of a given estimator provide valuable insights into its performance and highlight the theoretical limits of how accurately a parameter can be estimated given the available information. For parametric estimators, the Cramér-Rao lower bound provides the minimum possible variance of an unbiased estimator. In the nonparametric setting, establishing lower bounds is more challenging. Here, the variance of the efficient influence function acts as a nonparametric counterpart to the Cramér-Rao bound^^[[1]](#footnote-1)^^, characterising the best possible trade-off between bias and variance for estimating the target parameter[1]. The efficient influence function is a mathematical construct that quantifies the sensitivity of an estimator to small perturbations in the observed data. Formally, it is defined as the derivative of the estimator with respect to the underlying probability distribution of the data.

There are three main alternatives to build an influence-function based estimator[1]:

1. One-Step Estimator: this method adjusts bias in plug-in estimators by incorporating a correction term based on the efficient influence function. Using a correction term based on the efficient influence function, one-Step Estimator typically achieves desirable properties such as root-n consistency, asymptotic normality and efficiency under nonparametric conditions (such as sparsity or smoothness), even in complex or flexible modelling scenarios.
2. Estimating Equations: In this alternative approach, bias correction occurs at the distribution scale rather than the parameter scale. Bias is mitigated by setting the empirical mean of the efficient influence function to zero in an estimating equation. By doing so, bias is corrected in a way that adjusts the distribution of the estimated data to match better the true distribution, rather than focusing solely on correcting bias in the estimation of specific parameters.
3. Targeted Maximum Likelihood Estimation (TMLE): TMLE corrects bias by constructing a fluctuated estimate of the underlying probability distribution, aiming to set to zero the efficient influence on the new estimate. This adjusted distribution is then used to construct more accurate estimators.

Each of these alternatives has its own characteristics and advantages, and the choice often depends on the specificities of the problem and the desired properties of the estimator.

To mitigate error induced by the use of nonparametric models with estimators, two main strategies are typically used: complexity restriction and sample splitting. While the former imposes constraints on the model complexity^^[[2]](#footnote-2)^^ (it requires avoiding commonly used methods, such as Lasso or RandomForest) the latter prevents overfitting through data splitting, without the need of complexity restrictions (avoiding “double-dipping”^^[[3]](#footnote-3)^^ the data).

Regardless of the approach used, a number of conditions must be met by the estimated influence curve, such as some convergence properties, smoothness conditions and control on the magnitude of error term[1].

**Reference**

[1] Kennedy, E. H. (2022). Semiparametric doubly robust targeted double machine learning: a review. arXiv preprint arXiv:2203.06469.

1. The bound states that the variance of any unbiased estimator for a smooth functional (functions of the model parameters θ that are differentiable with respect to θ. Examples of smooth functionals include means, variances) must be greater than or equal to the inverse of the variance of the score function, scaled by the square of the derivative of the functional with respect to θ. [↑](#footnote-ref-1)
2. Donsker condition [↑](#footnote-ref-2)
3. when the same data is used for both model fitting and evaluation, leading to overly optimistic performance estimates [↑](#footnote-ref-3)
